# Granulomatous interstitial nephritis in a treatment-naïve patient with ulcerative colitis

**DOI:** 10.1080/0886022X.2022.2053714

**Published:** 2022-03-21

**Authors:** Muner M. B. Mohamed, Josean Flores-Santiago, Sudhir Perincheri, Juan Carlos Q. Velez

**Affiliations:** Department of Nephrology, Ochsner Health System, New Orleans, LA, USA; Ochsner Clinical School, The University of Queensland, Brisbane, QLD, Australia; Department of Pathology, Yale School of Medicine, New Haven, CT, USA

Dear Editor,

Renal and urological involvement in IBD ranges from 4 to 23% and has been described both in Crohn disease (CD) and in ulcerative colitis (UC) [1]. The most frequent kidney-related manifestations in patients with IBD are nephrolithiasis, tubulointerstitial nephritis, glomerulonephritis and AA amyloidosis [2]. Granulomatous interstitial nephritis (GIN) is a rare condition, presents in between 0.5 and 0.9% of native renal biopsies [3]. In IBD, almost all of the reported cases of GIN were in the context of recent exposure to 5-aminosalicylic acid (5-ASA) [4], and biotherapies as anti-tumor necrosis factor (TNF) treatment [5,6], We encountered an unusual case of GIN unrelated to medications in a patient who was subsequently diagnosed with IBD. To the best of our knowledge, no similar case has been reported to date.

**Figure 1.**
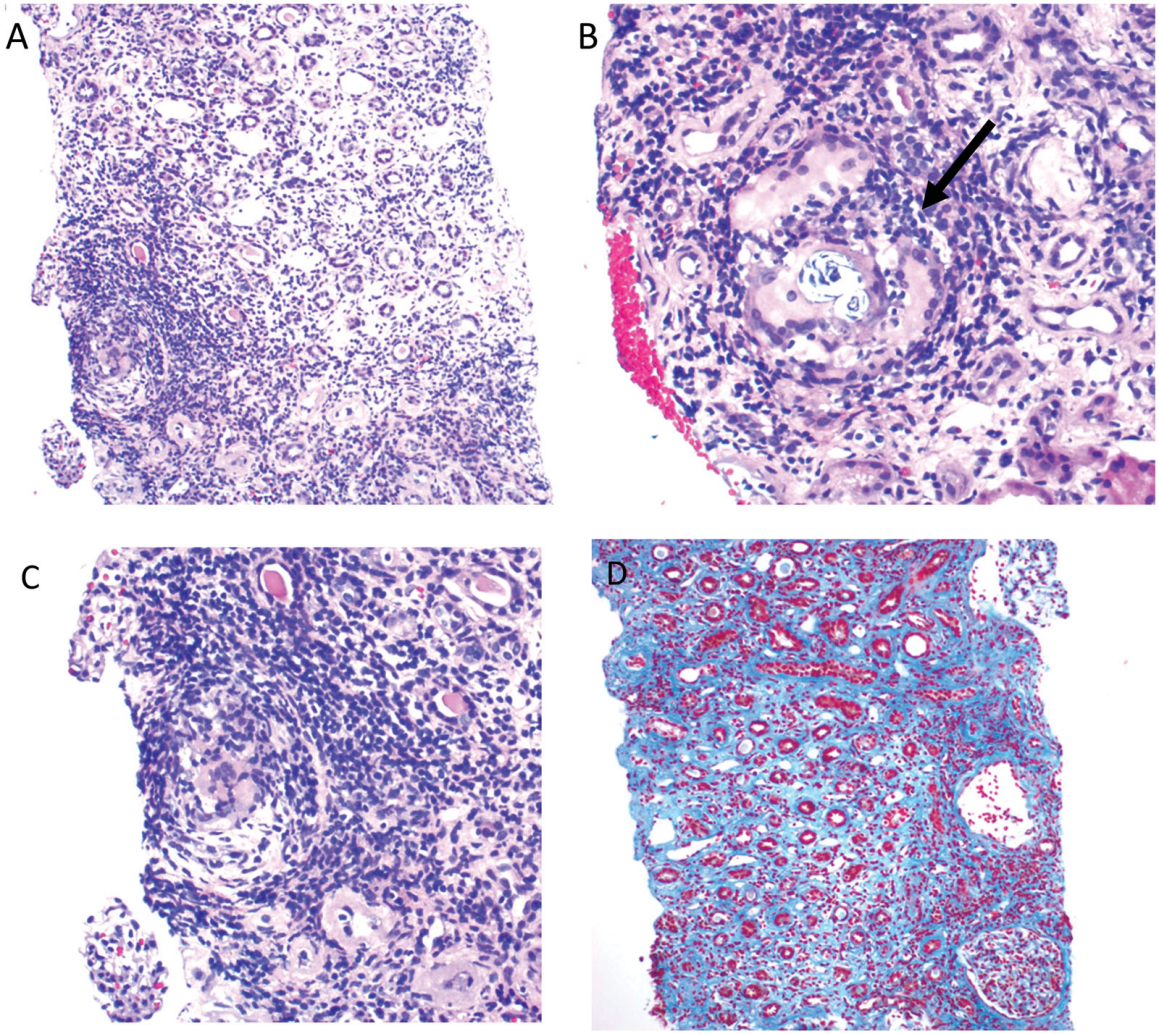
Light microscopic features of Granulomatous interstitial nephritis. Granuloma with Diffuse interstitial cellular infiltrate (A), granuloma with eosinophils in the surrounding inflammation ‘black arrow’ (B), zooming of the granuloma in image A (C), tubular atrophy and interstitial fibrosis (D). (hematoxylin-eosin, original magnifications ×100 [A] and ×200 [B&C]; Trichrome, original magnification ×100 [D].

A 24-year-old man was admitted to a nephrology clinic due to acute kidney injury. He had past medical history of allergic rhinitis. The patient denied any relevant family history. He had presented to another institute in a different city 2 weeks prior to evaluation with 4-month history of watery non-bloody diarrhea associated with abdominal pain, generalized malaise and unintentional weight loss. There was no report of fever, chills, night sweats, nausea, vomiting, change in appetite, hematuria or voiding difficulty. He was not taking any medications. Physical examination on presentation to the other facility revealed weight of 61.2 kg, normal blood pressure (122/75 mmHg), he was afebrile and not hypoxic; Physical examination was negative. He was admitted for diarrhea, noted to be in acute kidney injury (AKI) with a serum creatinine of 8.0 mg/dL that improved to 6.2 mg/dL with intravenous fluids (his baseline serum creatinine was 0.8 mg/dL documented 6 months prior). He received 2 units of packed red blood cell transfusion for acute anemia. The background of diarrhea was unclear. He had pyuria, but no significant proteinuria or hematuria. A kidney biopsy was performed on the 9th day of admission then he was discharged on day 10. He immediately flew back to New Orleans. Six days post discharge, the patient was seen at our nephrology clinic. He reported persistence of 2–3 watery non-bloody bowel movements daily and additional weight loss. He was normotensive, afebrile, his weight was down to 57.2 kg and physical examination was unremarkable. The laboratory data revealed persistence of AKI (Table 1). Chest radiograph did not show any acute cardiopulmonary process. Renal ultrasound showed mild increased echogenicity of the kidney`s parenchyma and no evidence of obstructive uropathy. Results of the kidney biopsy arrived 2 days later and revealed 29 glomeruli by light microscopy, one of which was globally sclerotic and one segmentally sclerotic. The specimen showed interstitial fibrosis with proportional atrophy involving 60–70% of the tissue, a non-caseating granuloma with diffuse interstitial infiltrate consisting of lymphocytes, plasma cells and occasional eosinophils involving 60% of the interstitium (Figure 1). Giant cell reaction is present surrounding several calcium phosphate deposits in the interstitium. The tubules showed proportional atrophy, acute tubular injury and tubulitis. The mesangium only showed mild hypercellularity. The vessels show focal mucoid edema. Tubular basement membrane was negative by immunofluorescence. Electron microscopy showed segmental thickening of the basement membrane with no subepithelial, subendothelial or intramembranous immunocomplex deposition. There was cytoplasmic vacuolization of the podocytes. The tubules show injury. There was a segmental podocyte foot process effacement (30–40%). Endothelial cells showed normal fenestrations. There were no subendothelial deposits. He was diagnosed with severe granulomatous interstitial nephritis with calcium phosphate deposition and severe interstitial fibrosis and tubular atrophy. The tissue was tested for tuberculosis (acid-fast bacilli) and was negative.

**Table 1.** Laboratory data at the time of nephrology clinic encounter.

Parameter	Normal Values	on admission 7/21/2020	First clinic visit 8/3/2020	3 weeks after steroids 9/3/2020	Last follow up 11/11/2020
*Clinical Chemistry*					
Sodium	136–145 mmol/L	136	140	138	143
Potassium	3.5–5.1 mmol/L	3.7	4.8	4.2	4.7
Chloride	95–110 mmol/L	110	103	107	106
Bicarbonate	23–29 mmol/L	16	22	17	25
Anion Gap	5–15 mmol/L	10	15	14	12
Blood urea nitrogen	6–20 mg/dL	85	95	114	52
Creatinine	0.5–1.4 mg/dL	7.86	7.2	4.8	4.6
eGFR	>60 mL/min/1.73 m2	9	9.6	15	17
Calcium	8.7–10.5 mg/dL	9	9.7	8.9	9.2
Glucose	70–110 mg/dL	89	79	83	91
Phosphorus	2.7–4.5 mg/dL		5.8	4.2	
Magnesium	1.6–2.6 mg/dL	1.9	1.9	1.4	2.2
Alkaline phosphatase	55–135 U/L	67	62		46
Protein total	6.0–8.4 g/dL	8.8	9.1		7.5
Albumin	3.5–5.2 g/dL	3.7	4.0	3.2	4.1
Bilirubin total	0.1–1.0 mg/dL	0.3	0.4		0.4
Bilirubin, direct	0.1–0.3 mg/dL		0.1		
AST	10–40 U/L	8	9		14
ALT	10–44 U/L	13	9		19
CRP	0.0–8.2 mg/L		26.9		
ESR	0–10 mm/Hr		29		
Vitamin D 1,25-Dihydroxy	20–79 pg/mL		28		
PTH	9.0–77.0 pg/mL		209		
*Complete blood count*					
Hemoglobin	14.0–18.0 g/dL	7.2	9.5	8.9	10.7
Platelets	150–350 K/uL	307	308	266	214
White cell count	3.90–12.70 K/uL	7.12	10.2	9.57	7.48
Neutrophils	38.0–73.0 %	57.9	67.6	80.3	73.4
Lymphocytes	18.0–48.0 %	22.6	16	8.9	17
Monocytes	4.0–15.0 %	14.7	1.2	9.5	7.1
Eosinophils	0.0–8.0 %	4.1	0.4	0.9	0.9
Basophils	0.0–1.9 %	0.6	0.07	0.1	0.3
*Iron/Anemia Profile*					
Iron	45–160 ug/dL		39		
TIBC	250–450 ug/dL		311		
Saturated iron	20–50 %		13		
Transferrin	200–375 mg/dL		210		
Folate	4.0–24.0 ng/mL		5.9		
Vitamin B-12	210–950 pg/mL		967		
*ACE*	16–85 U/L		24		
*Immunology*					
ANA Screen	Negative <1:80			Negative <1:80	
Anti-SSA Antibody	0.0–0.99 Ratio				
Anti-SSB Antibody	0.0–0.99 Ratio			0.05	
Complement (C3)	50–180 mg/dL			120	
Complement (C4)	11–44 mg/dL			42	
Streptolysin O Antibody	<200 IU/mL	297			
GBM antibody	<0.1 AI	<1.0			
*Infectious disease*					
Hepatitis B surface Ag	Negative			Negative	
COVID-19 PCR	Not Detected			Not Detected	
Tb Gold Plus	Negative			Negative	
Urine					
Color	Yellow, Straw, Amber	Straw		Straw	
Appearance	Clear	Clear		Clear	
Specific Gravity	1.005–1.030	1.009		1.010	
pH	5.0–8.0	5.0		6.0	
Glucose	Negative	Negative		Negative	
Protein	Negative	Negative		1+	
Ketones	Negative	Negative		Negative	
Occult blood	Negative	Negative		Negative	
Nitrite	Negative	Negative		Negative	
Urobilinogen	Negative	Negative		Negative	
Bilirubin	Negative	Negative		Negative	
Leukocytes	0–4 /hpf	Trace		Trace	
RBC	0–5 /hpf	2		1	2
WBC	None–Occ /hpf	14		34	1
Bacteria		occasional		Rare	Rare
protein/Creatinine ratio	0.00–0.20				0.17

ACE: Angiotensin converting enzyme; ANA: Antinuclear antibody; ALT: Alanine transaminase; AST: Aspartate transaminase; CRP: C-reactive protein; eGFR: Estimated glomerular filtration rate; ESR: erythrocyte sedimentation rate; GBM antibody: Anti–glomerular basement membrane antibody; Pco2: partial pressure of carbon dioxide; PCR: Polymerase Chain Reaction; PTH: Parathyroid hormone; RBC: red blood cell; TB: Tuberculosis; TIBC: Total iron binding capacity; WBC: white blood cell.

He began treatment with prednisone 60 mg daily and tapered down over the course of 3 months. After 3 weeks, serum creatinine improved from 7.2 to 4.8 mg/dL. Upper endoscopy and colonoscopy were done within 2 weeks of initiation of steroid therapy and revealed ulcerative colitis with moderate-to-severe pancolitis. Adalimumab was added by a gastroenterologist. Prednisone was tapered down and azathioprine was added for maintenance. Three months later, his kidney function stabilized with a serum creatinine of 4.6 mg/dL and gastrointestinal symptoms improved at last follow up visit.

IBD is associated with a spectrum of kidney complications related to either chronic inflammation or drug therapy [4]. As in other extraintestinal manifestations, kidney involvement can be considered as being related to the same immunological mechanisms that cause the intestinal inflammation, to metabolic disorders that develop in IBD, or to drug-induced toxicity [2]. The most common renal involvement found in kidney biopsy is immunoglobulin A nephropathy, followed by interstitial nephritis [4]. In one study of 83 patients with IBD who underwent kidney biopsy, 5 were found to have GIN. All of the cases of GIN in that report had a history of current or recent past exposure to amino-salicylates [4]. This drug-induced nephrotoxicity is thought to represent an idiosyncratic, delayed-type hypersensitivity reaction that is independent of dose and duration of exposure [7]. With the arrival of newer therapies, amino-salicylates are no longer a common therapy in IBD. Rare cases of GIN associated with anti-TNF therapy have emerged [6–8].

GIN in therapy-naïve patients with IBD is extremely rare. To date, only 6 case reports of therapy-naïve patients with Crohn disease developing GIN have been described in the English literature [8], whereas no prior case of therapy-naïve UC developing GIN has been reported.

The calcium phosphate deposits identified in the biopsy likely correspond to hyperphosphatemia and an alkaline urine pH (possibly from exogenous bicarbonate to treat metabolic acidosis) during the early stage of the acute kidney injury. The focal areas of podocyte injury likely correspond to glomerular maladaptive changes secondary to the severe tubulointerstitial lesion. Altogether, the calcium phosphate deposition and the focal podocytopathy likely contributed to the accelerated progression of CKD.

Despite the severity of the pathological and laboratory findings, the patient had a partial response to immunosuppression. In a retrospective study of 18 patients with GIN with different underlying etiologies, treatment with a moderate dosage of prednisolone was associated with a good prognosis (relative to other causes of renal failure) irrespective of the underlying cause and of the degree of interstitial fibrosis [9]. In an English literature review of 22 patients with UC and mesalazine-induced interstitial nephritis, 61% had residual chronic kidney disease and 13% developed end-stage kidney disease [7].

In summary, we submit that GIN should be considered as a rare but potential extraintestinal manifestation of IBD that can present as AKI. Close monitoring kidney function in patients with IBD even among those not treated with mesalazine.
